# Arctic avian predators synchronise their spring migration with the northern progression of snowmelt

**DOI:** 10.1038/s41598-020-63312-0

**Published:** 2020-04-29

**Authors:** Teja Curk, Ivan Pokrovsky, Nicolas Lecomte, Tomas Aarvak, Kurt Burnham, Andreas Dietz, Alastair Franke, Gilles Gauthier, Karl-Otto Jacobsen, Jeff Kidd, Stephen B. Lewis, Ingar J. Øien, Roar Solheim, Karen Wiebe, Martin Wikelski, Jean-François Therrien, Kamran Safi

**Affiliations:** 1 Max Planck Institute of Animal Behavior, Department of Migration, Am Obstberg 1, Radolfzell, 78315 Germany; 2 University of Konstanz, Department of Biology, Universitätsstraße 10, Konstanz, 78464 Germany; 3 Institute of Plant and Animal Ecology, Ural Division Russian Academy of Sciences, 8 marta str. 202/3, Yekaterinburg, 620144 Russia; 4 Institute of Biological Problems of the North, Magadan, Ulitsa Portovaya 18, 685000 Russia; 5 Canada Research Chair in Polar and Boreal Ecology, Department of Biology, Université de Moncton, 18 Antonine-Maillet, Moncton, NB E1A 3E9 Canada; 6 Norwegian Ornithological Society, BirdLife Norway, Sandgata 30B, Trondheim, 7012 Norway; 7 High Arctic Institute, 603 10th Avenue, Orion, IL 61273 USA; 8 German Aerospace Center (DLR), German Remote Sensing Data Center (DFD), Pfaffenwaldring 38-40, Stuttgart, 70569 Germany; 9 University of Alberta, Faculty of Science, 116 St NW, Edmonton, AB T6G 2R3 Canada; 10 Université Laval, Department of Biology and Centre d’études nordiques, 1045 avenue de la Médecine, Québec, QC G1V 0A6 Canada; 11 Norwegian Institute for Nature Research, Department of Arctic Ecology, Hjalmar Johansens gate 14, Tromso, 9296 Norway; 12 Kidd Biological Inc, 2911 Meridian Court, Anacortes, WA 98221 USA; 13 U.S. Fish and Wildlife Service, Division of Migratory Bird Management, 3000 Vintage Blvd 201, Juneau, AK 99801 USA; 14 University of Agder, Zoological Department, Universitetsveien 25 D, Kristiansand S, 4630 Norway; 15 University of Saskatchewan, Department of Biology, 112 Science Place, Saskatoon, S7N 5E2 Canada; 16 Hawk Mountain Sanctuary, Acopian Center for Conservation Learning, 410 Summer Valley Road, Orwigsburg, PA 17961 USA

**Keywords:** Animal migration, Behavioural ecology, Boreal ecology, Climate-change ecology, Ecological modelling

## Abstract

Migratory species display a range of migration patterns between irruptive (facultative) to regular (obligate), as a response to different predictability of resources. In the Arctic, snow directly influences resource availability. The causes and consequences of different migration patterns of migratory species as a response to the snow conditions remains however unexplored. Birds migrating to the Arctic are expected to follow the spring snowmelt to optimise their arrival time and select for snow-free areas to maximise prey encounter en-route. Based on large-scale movement data, we compared the migration patterns of three top predator species of the tundra in relation to the spatio-temporal dynamics of snow cover. The snowy owl, an irruptive migrant, the rough-legged buzzard, with an intermediary migration pattern, and the peregrine falcon as a regular migrant, all followed, as expected, the spring snowmelt during their migrations. However, the owl stayed ahead, the buzzard stayed on, and the falcon stayed behind the spatio-temporal peak in snowmelt. Although none of the species avoided snow-covered areas, they presumably used snow presence as a cue to time their arrival at their breeding grounds. We show the importance of environmental cues for species with different migration patterns.

## Introduction

Animals are expected to alter their behaviour as a response to ongoing climate change^1^. Animal movement is a behaviour that can be strongly influenced by external, biotic and abiotic environmental factors^2^. Several studies suggested that avian species adjust their movements in response to environmental conditions^3–5^ and consequently, optimise their reproduction and survival^6,7^. However, more evidence is necessary to fully understand this mechanism. It is thus important to study individual and species-specific movement responses to the environment, and assess whether these responses differ regarding their spatio-temporal scale^8^.

Avian migration i.e. seasonal movement between breeding and non-breeding areas is in large part driven by the availability of resources^9^. Furthermore, the predictability of resources is presumed the main stimulus of migration pattern^10,11^. Ephemeral resources, unpredictable in time and space, can lead to irruptive (facultative) migration, whereas predictable availability of resources is associated with regular (obligate) migration. However, species and populations can display either of these two migration patterns or a mixed migration pattern that combines elements of both behaviours. Depending on the predictability of available resources, arctic-breeding raptor species exhibit contrasting migration patterns, representing a gradient from irruptive, mixed, to regular migration. The snowy owl *(Bubo scandiacus)* is irruptive in parts of its range with a variable migration schedule, and weak fidelity to breeding sites^10,12–14^. The rough-legged buzzard *(Buteo lagopus)* exhibits some flexibility in its migration schedule and site fidelity, but it can occasionally show irruptive movements^15^. The peregrine falcon *(Falco peregrinus)* is a regular migrant that has a fixed migration schedule in combination with high breeding-site fidelity^16–18^.

Migration patterns in birds are among other factors linked to the predictability of available prey and consequently diet of the species^7,19^. Because prey availability and abundance fluctuates across space and time and differently among prey species^19^, diet generalists have a greater chance than specialists to encounter at least some prey types. During the breeding season, the snowy owl is a specialist feeding mostly on microtine rodents^20^, a highly fluctuating food resource^12^. The rough-legged buzzard has a mixed diet, specialising on small mammals when available, switching to medium-sized mammals and birds when small mammals are scarce^20–22^. The peregrine falcon is a diet generalist, feeding on medium-sized birds, a non-fluctuating resource, but also on small mammals^20,23^. Prey availability for the peregrine falcon (a generalist predator) should thus be less variable among years than for the snowy owl (specialist predator) with the rough-legged buzzard in between. While in both irruptive and regular patterns food availability is the ultimate cause for migration, in irruptive migrants it also acts as a proximate stimulus. Irruptive migrants thus respond to local food conditions directly by delaying/advancing migration^11^, whereas regular migrants should rely less on food conditions but more on proxies such as day length change coupled to a strong endogenous control^24^. The proximate response is especially important during spring migration for migratory species breeding in the Arctic. The window of opportunity with favourable environmental conditions for successful breeding in the Arctic is very narrow and the start of breeding is restricted to a few days only. Arriving at the breeding grounds at the right time is thus crucial for successful reproduction^25–27^. While early arrival secures a larger time window for breeding, being too early can expose migrants to harsh weather conditions and low food resources. Migrants should thus adjust their movement during spring migration to arrive at the breeding grounds at the optimal time.

Snow cover can limit access to small mammals at the breeding grounds^28,29^. For this reason, arctic migrants may follow the northern progression of snowmelt during spring migration to optimise arrival time. The snowy owl, being mainly an irruptive migrant and lemming specialist, is expected to closely follow the progression of snowmelt, followed by the rough-legged buzzard and then the peregrine falcon. Arctic migrants may also actively avoid snow-covered areas because of the limited access to small mammals^30^ when they feed during migration^31^. The snowy owl, which relies most strictly on small mammals may exhibit the strongest preference towards snow-free areas, somewhat lower preference is expected from the rough-legged buzzard and the lowest preference from the peregrine falcon. The literature on this topic is scarce (but see^32,33^) and the process of decision-making during migration as a response to snow conditions is largely unexplored. Other environmental factors such as temperature, day length^34^, productivity (NDVI - normalized difference vegetation index)^35^ and wind conditions^36,37^ were previously described influencing arctic migrants. Here we focus on snow cover as an additional factor that was rarely investigated before and might modulate spring migration movements.

In this study, we assess the impact of snow cover on movements during the spring migration of three top predator species of the tundra. We explore whether they respond differently to the snow cover depending on migration pattern (i.e., irruptive vs regular). First, we investigate whether arctic migrants follow the northern progression of snowmelt and whether the irruptive and regular migrants respond to the snowmelt progression differently. Second, we examine whether they avoid snow-covered areas from alternative options in the environment during the spring migration. We also test whether the avoidance of snow-covered areas differs with the spatio-temporal scale. We predict that (1) irruptive migrants follow the snowmelt the most, regular migrants least, and the effect of snowmelt on migratory movements of mixed migrants should be intermediate; (2) arctic species avoid areas with snow cover, with the irruptive migrants showing the highest and the regular migrants the lowest avoidance, and mixed migrants exhibiting intermediate avoidance.

## Methods

### Movement data

**Figure 1:**
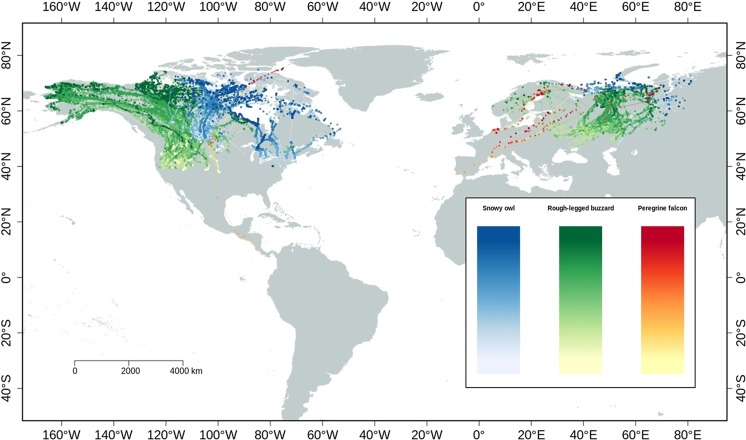
Spring migration tracks of the snowy owl, rough-legged buzzard, and peregrine falcon (2001–2018). Colour gradients from lighter to darker tones represent spring dates of migration periods, from earlier to later date respectively.

We used Argos and GPS transmitters deployed from 2001 to 2018 in North America and Eurasia to collect data on three arctic species. We confirm that all experimental protocols were officially approved and all methods were carried out in accordance with the relevant guidelines and regulations. The following boards approved our study: University of Saskatchewan, Animal Protection Committee of Laval University, Animal welfare unit in Norway, Norwegian Environment Agency, USGS Bird Banding Lab, Government of Greenland and The Danish Polar Center. Details about permits, capture methods^38^, sites and transmitters used are provided in the supplementary material. We removed Argos locations with CLS Argos class lower than 2 (error >500 meters)^39^. We did not filter GPS locations because of their high accuracy (<100 meters). Since we were specifically interested in spring movement, we extracted spring migration tracks using First Passage Time (see “Determination of spring migration periods” in the supplementary material). After data cleaning, we used 225 individuals that bred in the Arctic for analyses of spring migration, totalling in 245,509 spatial points (Table 1, Fig. 1). Since data were collected as parts of several projects, sampling intervals were not consistent (min = 1 second, median = 6 minutes, max = 39 days). We dealt with these differences by using Step Selection Function (SSF) so that the consecutive positions were separated by a certain threshold value (see below).

**Table 1:** Summary of species, individuals, tracks, days, locations and sampling frequencies included in the study.

Species	Number of ind.	Number of spring migr. tracks	Number of days per track (mean ± SD)	Number of locs per track (mean ± SD)	Sampling freq. (min, median, max)
Snowy owl	98	211	37 ± 35	403 ± 1008	1 second, 31 minutes, 36 days
Rough-legged buzzard	112	219	72 ± 54	727 ± 766	1 second, 2 minutes, 39 days
Peregrine falcon	15	18	32 ± 21	76 ± 81	1 second, 8 minutes, 22 days

### Data analyses

#### Environmental variables

We obtained daily snow cover data with a spatial resolution of 500 meters (Global SnowPackMODIS) from the German Aerospace Center (DLR). The obtained snow-covered data matched the extents and dates of spring migration tracks of the three species (see supplementary material under “Determination of spring migration periods”). This product is based on the daily snow cover products MOD10A1 and MYD10A1 (version 5 and 6 as provided by the National Snow and Ice Data Center NSIDC) of the Moderate Resolution Imaging Spectroradiometer (MODIS), which has been reprocessed to remove the effects of cloud cover and polar darkness^40^. Note that after the interpolation process the information about snow cover fraction is lost as only algorithm for interpolating binary snow cover is currently available. Using the R package “raster”^41^, we annotated snow cover values to the modelled movement tracks linking individual locations with snow cover data from the same dates. We calculated day length (hours) using the “daylength” function from the “geosphere” R package^42^. Through the open-source Env-DATA system^43^ we obtained and annotated the following environmental variables: air temperature (°C) at a resolution of 6 hours and 0.75 degrees (ECMWF interim full daily SFC, 2 meters above ground), vegetation index (NDVI) with a resolution of 0.05 degrees and 16 days (MOD13C1) and wind direction variables (U, the east-west component of the wind; V, the north-south component of the wind) with a resolution of 6 hours and 0.75 degrees (ECMWF interim full daily at surface, 10 meters above ground). Based on the U and V components (meters per second), we calculated wind support (wind in the direction of each bird’s movement) and crosswind (wind perpendicular to the bird’s movement)^44^, where heading and ground speed were derived from the locations of the individual track.

#### Step Selection Function

We used Step Selection Function (SSF) to investigate how environmental predictors affect spring movements of arctic raptors^45–48^. The method assumes discrete movement decisions, represented by a fixed time step length. At each step, an animal chooses a location out of a set of available alternate locations characterised by differences in environmental conditions fitted to a conditional logistic regression. We modelled SSFs separately for species and step lengths of one day, three days, and five days to investigate whether decision-making during spring migration changes at different temporal scales (“amt” R package^49^). Step lengths were chosen so that no auto-correlation of environmental data in time is present (see lags for one - five days in Supplementary Fig. S1). We readjusted the data according to the chosen step length so that when two observations were separated by less than the step length value, the second observation was removed, and when two observations were separated by more than the step length value, the later observation was assigned to a different burst. A burst is a segment of a track that includes only locations separated by the chosen step length value. We estimated the distribution of alternative steps using distance and relative angle (angle between the previous and new direction of movement) of the chosen steps within individual migration tracks of each species. Using an exponential distribution, we randomly selected 10 alternative locations for each actual chosen location. These alternative locations together with a chosen location at each step form a stratum. Finally, we annotated chosen and alternative locations with the relevant environmental variables.

#### Spring movements in relation to the northern progression of snowmelt

**Figure 2:**
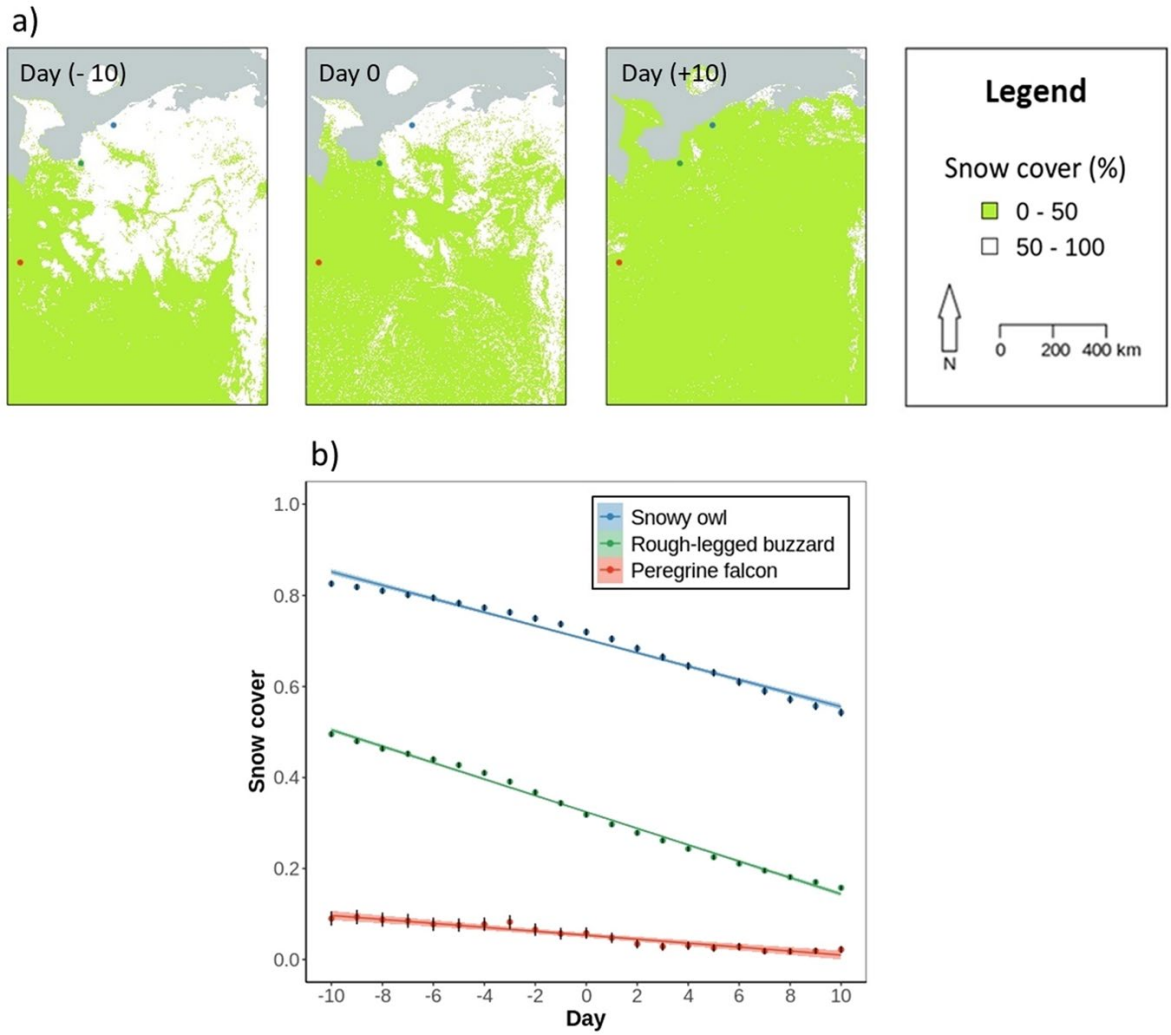
(**a**) Snow cover conditions at the location of snowy owl (blue dot), rough-legged buzzard (green dot), and peregrine falcon (red dot) in 2016 on 2 May (10 days in the past), 12 May (current), and 23 May (10 days in the future). (**b**) Snow cover conditions (0 = snow absence; 1 = snow presence) at each of the birds’ position compared between the days (from 10 days in the past to 10 days in the future) (Table 2). Dots with lines represent mean ± SE of the raw data and shaded areas represent SE of the model estimates.

We used the chosen locations of individuals at a one-day step length to investigate whether arctic migrants followed the spring snowmelt. We independently tested day length, temperature and NDVI to evaluate whether these factors additionally influence spring movements in arctic migrants. At each location, we extracted snow cover, day length, temperature and NDVI values and compared them between the past, present, and future (from 10 days in the past to 10 days in the future), separately for each species (Fig. 2a). The scale of 10 days was chosen to exclude possible auto-correlation of environmental data in time (Supplementary Fig. S1). Note also that using larger (15 and 20 days) or smaller (5 days) time-windows resulted in the same overall effect (Supplementary Fig. S2). We used generalised linear mixed models (GLMM) implemented in “lme4”^50^ R packages. In the model, we used snow cover, temperature, day length and NDVI as dependent variables (separately in each model), day as a predictor variable, and year nested in individual as a random effect. We checked the assumptions of the tests and compared models containing all predictors with models leaving out specific predictors to evaluate the effect of each predictor, using Akaike’s information criterion (AIC). We considered models with an ΔAIC > 2 as different where the model with lower AIC has stronger support^51^.

#### Movement decisions based on snow cover

**Figure 3:**
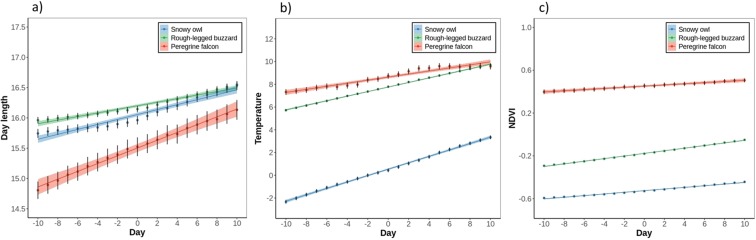
(**a**) Day length (hours), (**b**) temperature (°C), (**c**) NDVI (vegetation index from −1 to 1) at each of the birds’ position compared between the days (from 10 days in the past to 10 days in the future) (Table 2). Dots with lines represent mean ± SE of the raw data and shaded areas represent SE of the model estimates.

We compared environmental conditions between chosen and alternative locations (Fig. 3a) to test whether arctic migrants avoided snow-covered areas when following the receding snow line. In the analysis, we also included wind support and crosswind (only for one-day step length) to test whether these factors would influence the birds’ choice of distance and direction and to evaluate whether snow cover predicted movement decisions irrespective of the wind conditions. We fitted mixed conditional logistic regression models using “coxme” function from “coxme” R package^52,53^, separately by species and separately for one-, three-, and five-day step lengths. As a dependent variable, we included movement decision (chosen vs alternative), as predictors, snow cover, wind support, and crosswind, and as a random effect stratum nested in individual. We performed Shapiro-Wilk’s tests for multivariate normality (p < 0.001 for each species) and check the test’s assumptions. Correlation coefficients between snow cover, wind support, and crosswind were less than 0.7^54^, thus we included these variables in the same model. Based on the Akaike’s information criterion (AIC), we compared the full models with those without the predictor of interest to evaluate the effect of this predictor and select a better model. We considered models with an ΔAIC > 2 as different, with lower AIC having more support^51^. We validated the models with Used habitat calibration (UHC) plots^55^ by comparing the distribution of the observed and predicted values of explanatory variables at the chosen locations with the distribution at alternative locations.

## Results

### Spring movements in relation to the northern progression of snowmelt

All three species followed the northern progression of snowmelt but responded to snowmelt progression differently. Movements of the rough-legged buzzards were most tightly adjusted to snow progression, those of the snowy owl less, and those of the peregrine falcon the least (see coefficients, SE’s and ΔAIC in Table 2 and in Supplementary Table S1 and difference in the slopes in Fig. 2). Higher temperature, longer days and higher NDVI were positively correlated with the spring movements in all three species (see ΔAIC in Table 2 and Fig. 3). For the owl, temperature was the most important factor, following by snow cover, for the buzzard, snow cover was the most important followed by NDVI and for the falcon, temperature was the most important, followed by day length (see ΔAIC in Table 2). The species also greatly differed in the snow conditions in the areas they used during spring migration. The owl occurred mostly in snow-covered regions, the buzzard used partially snow-covered areas, and the falcon used snow-free areas (see the snow cover values at day zero; Fig. 2). This can be seen also with individual migration profiles where responses to snow cover were detected (Supplementary Fig. S3). The owl experienced little changes between snow-free and snow-covered areas during migration and continued moving north after entering a snow-covered area. The falcon also experienced little changes between snow-covered and snow-free areas but migrated only when the area was free of snow. The buzzard experienced high variability in the snow-covered and snow-free areas and when it reached the snow-covered area during migration, it waited or retreated to lower latitude before continuing its northward migration.

**Table 2:** Snow cover, day length, temperature and NDVI values extracted at each location during spring (at one-day step length) and compared between days (from 10 days in the past to 10 days in the future), separately for each species.

Species (n = number of locations)	Dep. var.	Pred.	Est.	SE	z/t value	p value	ΔAIC	AIC Weight	LL
Snowy owl n = 67572	Snow cover	(Intercept)	1.70	0.51	3.35	<0.001	3461*	1.0	−30794
		Day	−0.10	0.00	−56.70	<0.001			
	Day length	(Intercept)	15.44	0.37	41.64	<0.001	446*	1.0	−181330
		Day	0.05	0.00	21.21	<0.001			
	Temperature	(Intercept)	0.82	1.23	0.67	0.51	4543*	1.0	−224904
		Day	0.30	0.00	68.57	<0.001			
	NDVI	(Intercept)	−0.40	0.05	−7.70	<0.001	3420*	1.0	2472
		Day	0.01	0.00	59.25	<0.001			
Rough-legged buzzard n = 133461	Snow cover	(Intercept)	−1.04	0.15	−7.00	<0.001	8186*	1.0	−72895
		Day	−0.10	0.00	−87.11	<0.001			
	Daylength	(Intercept)	15.44	0.23	67.46	<0.001	501*	1.0	−366250
		Day	0.04	0.00	22.44	<0.001			
	Temperature	(Intercept)	7.07	0.44	15.93	<0.001	6314*	1.0	−417478
		Day	0.20	0.00	80.43	<0.001			
	NDVI	(Intercept)	−0.22	0.02	−11.16	<0.001	7321*	1.0	−42750
		Day	0.01	0.00	86.76	<0.001			
Peregrine falcon n = 6575	Snow cover	(Intercept)	−3.90	1.08	−3.61	<0.001	134*	1.0	−899
		Day	−0.13	0.01	−10.90	<0.001			
	Day length	(Intercept)	16.66	0.83	20.04	<0.001	185*	1.0	−14843
		Day	0.06	0.00	13.76	<0.001			
	Temperature	(Intercept)	6.65	1.76	3.78	0.007	299*	1.0	−17929
		Day	0.13	0.01	17.56	<0.001			
	NDVI	(Intercept)	0.13	0.21	0.63	0.55	107*	1.0	−1079
		Day	0.01	0.00	10.46	<0.001			

Generalised linear mixed models (GLMM) with snow cover, day length, temperature and NDVI as dependent variables (each in a separate model), day as a predictor variable, and year nested in individual as a random effect. To evaluate the effect of the predictor, we compared a model with and without that predictor. Only the results of the full models are presented and those with ΔAIC > 2 are marked with *.

### Movement decisions based on snow cover

**Figure 4:**
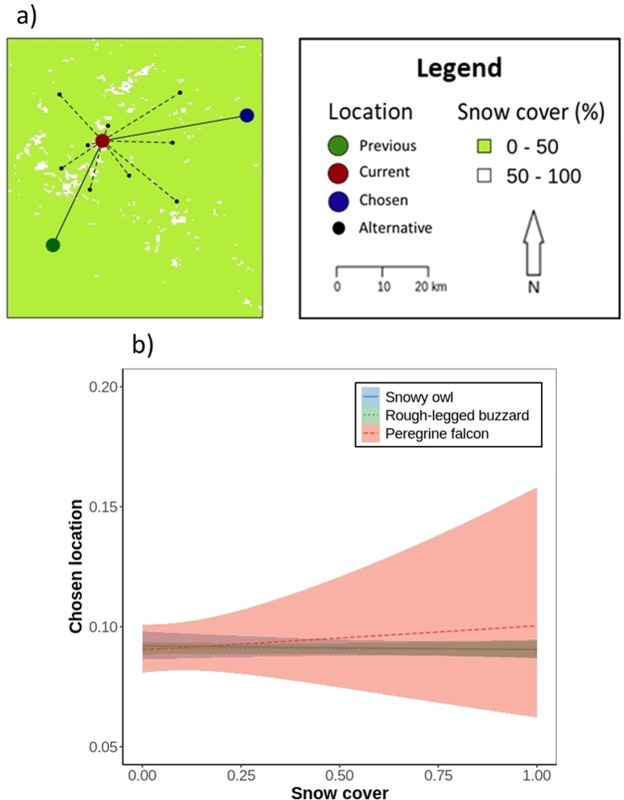
(**a**) An example of the stratum in the step selection function with chosen and alternative locations and the snow cover background. (**b**) The impact of snow cover (0-snow absence, 1-snow presence) on movement preference (0-alternative vs 1-chosen locations) of arctic raptors during the spring migration at steps separated by one day (Table 3). Shaded areas represent SE of the model estimates.

The three species did not exhibit any preference or avoidance for snow cover during spring migration when assessing movement decisions at one-day steps (see coefficients, SE’s and ΔAIC values on Table 3, and slopes on Fig. 4). At three- and five-day steps, no response to snow cover remained for the three species (see ΔAIC values in Supplementary Table S1). Wind conditions measured at one-day steps, however, influenced their movement decisions. The owl and the falcon moved towards locations with less wind support and the buzzard towards locations with more wind support and crosswind (see ΔAIC values on Table 3, and slopes on Supplementary Fig. S4). Model validation confirmed the significant effects of wind support and crosswind (see UHC plots in Supplementary Fig. S5).

**Table 3:** Movement decisions of arctic raptors according to environmental predictors (snow cover, wind support, and crosswind) at one-day step length.

Species (n = number of locations)	Pred.	Coef.	SE	z value	p value	ΔAIC	AIC Weight	LL
Snowy owl n = 41316	Snow cover	−0.01	0.04	−0.13	0.90	2	0.3	−35026
	Wind support	−0.05	0.00	−10.99	<0.001	118*	1.0	
	Crosswind	−0.00	0.01	−0.52	0.61	2	0.3	
Rough-legged buzzard n = 72479	Snow cover	−0.05	0.03	−1.88	0.06	2	0.7	−72409
	Wind support	0.01	0.00	2.92	0.004	6*	1.0	
	Crosswind	0.06	0.00	17.02	<0.001	282*	1.0	
Peregrine falcon n = 4147	Snow cover	0.07	0.25	0.29	0.77	2	0.3	−2753
	Wind support	−0.06	0.02	−3.53	<0.001	10*	1.0	
	Crosswind	0.03	0.02	1.78	0.08	1	0.6	

We performed mixed conditional logistic regression models with movement choice (chosen vs alternative locations) as a dependent variable, snow cover, wind support, and crosswind as predictors and stratum nested in individual as a random effect. We performed the models separately by species. To evaluate the effect of the predictor, we compared models with and without that predictor. Only the results of the full models are presented and those with lower AIC and ΔAIC > 2 are marked with *.

## Discussion

Consistent with our first prediction, spring migratory movements of all three arctic raptors we studied were synchronised with the northern progression of snowmelt. In addition to temperature, photoperiod and NDVI, the arctic raptors presumably use also snow cover as a cue during spring migration to avoid arriving too early or too late at the breeding grounds. Indeed, snow cover was the most important predictor for the buzzard, less for the owl and least for the falcon when comparing snow cover with temperature, day length and NDVI. Optimising the timing of arrival allows migrants to find territories rich in resources and to start breeding when conditions are favourable^56,57^. However, despite the overall trend in following snowmelt progression, our species differed in the details of their responses. We predicted that irruptive species (snowy owl) would track snowmelt most closely, mixed migrants (rough-legged buzzard) would have a weaker association, and that regular migrants (peregrine falcon) would show little association with snowmelt. Consistent with this, the owl and the buzzard were more flexible and followed the snowmelt more than the falcon but surprisingly, the owl responded to the progression of snowmelt less than the buzzard. Perhaps the owl followed the progression of snowmelt by moving to different directions rather than with directed movements. Thus, the pattern of following the northern progression of snowmelt was less evident in the owl than in the buzzard. There is indeed evidence that snowy owls visit and search for suitable areas to settle and breed during spring migration^12^.

While moving to northern breeding sites, the species occupied areas with different snow coverage and thus seemed to follow the northern progression of snowmelt differently. The owl returning to northern breeding areas moved between areas with 75% of snow cover, therefore was ahead of snowmelt. The slow speed of snowmelt in areas the owl passes during the migration can explain our result of an apparent tighter response in the buzzard than in the owl. The buzzard which moved between areas with 35% of snow cover was at the peak of snowmelt, passing at the time when snowmelt was most rapid. Indeed, the melt of snow likely follows a sigmoidal curve, slow at first, rapid at 50% snow cover and slow at the end when only patches of snow remain^58^. The buzzard also experienced many changes between snow-free and snow-covered areas which confirms the species being on the peak of snowmelt. In addition, the progression of the buzzard’s northern movements was strictly limited by the snow cover in comparison to the other two species which is another reason for the apparent strongest response to snowmelt in this species. The falcon mostly occupied areas with 10% snow cover and was thus behind the peak of snowmelt. This outcome corresponds to the result that the falcon showed the smallest response to the progression of snowmelt.

Contrary to our second prediction, arctic raptors neither showed a preference nor avoidance for snow-covered areas when following the receding snow line. We predicted that arctic species avoid areas with snow cover, with the owl showing the highest avoidance, the buzzard intermediate avoidance, and the falcon the lowest avoidance. One reason for no response to snow cover could be that the snow cover can represent different conditions of food availability depending on the circumstances. As snow cover affects food availability, the snowy owl and the rough-legged buzzard, which rely on small mammals for food^20^, can find prey on snow-free areas^59^. Snow-covered areas might be preferable, given that small mammals are known to concentrate under the snow^28,29,59,60^. As the spring progresses and the snow starts melting, small mammals may not be concentrated under the snow but dispersed, thus the timing in spring could also play a role. Also, physical properties of snow such as depth and density need to be considered when investigating the availability of small mammals in spring^28,60^. In addition, there might be species-specific differences in the preference or avoidance of snow-covered areas depending on their thermoregulation and camouflage requirements^61^.

Other environmental factors additionally influenced the movements of arctic raptors. Besides the northern progression of snowmelt, raptors synchronised their spring migration movements with the increasing temperature, longer days and higher productivity. These environmental factors influenced spring migration timing in several northern migrants, for example, the pink-footed goose *(Anser brachyrhynchus)*^34^ and the barnacle goose *(Branta leucopsis)*^62^. When investigating movement preference at each step during the spring migration, movement decisions based on wind conditions differed between the species. The owl and falcon moved towards areas with less wind support while the buzzard moved towards more wind support and crosswind. These results imply that arctic raptors are not necessarily negatively affected by wind support and crosswind and might compensate for the drift similar to the honey buzzard *(Pernis apivorus)*^63^. Wind conditions, thus, seemed to play a secondary role in movement decisions of arctic raptors.

Our study demonstrates that spatio-temporal changes in snow cover affect the movements of arctic raptors depending on the species differently. The study provides evidence that the species breeding in the same environment and exhibiting contrasting migration patterns respond differently to the snow conditions. Rapid environmental change such as a global increase in temperature is expected to shape snow patterns^64^ resulting in more rapid snowmelt and prolonged snow-free periods^65^. With earlier greening, some species might have broader windows of opportunity for breeding. However, environmental conditions are forecasted to become less predictable too^66^ which represent a challenge for these species. Resulting spatio-temporal changes in food availability^67,68^ could force species to alter migration patterns or even strategies^69,70^. The ability of a species to adjust movements to environmental conditions likely depends on the degree of phenotypic plasticity^71,72^. The peregrine falcon, having regular (less flexible) migration would have probably the most difficulty adjusting to changing conditions, which can result in a phenological mismatch^73,74^ affecting fitness. In contrast, the snowy owl and the rough-legged buzzard which have more flexible migration, are expected to more easily adapt to these changes.

Changing snow cover patterns caused by climate change will likely shape not only migration patterns of species but have effects that cascade through the entire ecosystem and shape a species’ phenology, breeding success, and survival. Thus, at a larger scale, population dynamics, species distribution, and trophic interactions will likely also change^75^. These changes might occur especially rapidly in the Arctic ecosystem where warming was three times greater than elsewhere over the last three decades^66,76^. Studying how species track and use the environment is therefore of great importance to predict the impact of future changes. The next step is to model how migration patterns change under different climate scenarios and identify the species in need of management actions. We speculate that our results are applicable to all other irruptive, regular and intermediate migrants in the Arctic but detailed data on the migration patterns of more species are needed to test the applicability of our results. Species might show different responses to the snow cover not only as a result of migration pattern but also due to differences in migration timing and distance, diet, and possibly physical and physiological adaptation to snow. Although we could not tease apart the potential effects of such variables here, we were able to provide one of the most robust comparisons among species breeding in the same environment. Our study is thus an important step towards understanding the proximate cues that species with different migration patterns use in response to environmental conditions.

## Supplementary information

Supplementary material.

## Data Availability

All datasets used in this study are available upon request from the authors.
