# Author Correction: Arctic avian predators synchronise their spring migration with the northern progression of snowmelt

**DOI:** 10.1038/s41598-020-72948-x

**Published:** 2020-09-17

**Authors:** Teja Curk, Ivan Pokrovsky, Nicolas Lecomte, Tomas Aarvak, David F. Brinker, Kurt Burnham, Andreas Dietz, Andrew Dixon, Alastair Franke, Gilles Gauthier, Karl-Otto Jacobsen, Jeff Kidd, Stephen B. Lewis, Ingar J. Øien, Aleksandr Sokolov, Vasiliy Sokolov, Roar Solheim, Scott Weidensaul, Karen Wiebe, Martin Wikelski, Jean-François Therrien, Kamran Safi

**Affiliations:** 1 Department of Migration, Max Planck Institute of Animal Behavior, Am Obstberg 1, 78315 Radolfzell, Germany; 2 Department of Biology, University of Konstanz, Universitätsstraße 10, 78464 Konstanz, Germany; 3 Arctic Research Station of Institute of Plant and Animal Ecology, Ural Division Russian Academy of Sciences, 21 Zelenaya Gorka Str., Labytnangi, Yamal-Nenets Autonomous District, Russia 629400; 4 Institute of Biological Problems of the North, Ulitsa Portovaya 18, Magadan, Russia 685000; 5 Canada Research Chair in Polar and Boreal Ecology, Department of Biology, Université de Moncton, 18 Antonine-Maillet, Moncton, NB E1A 3E9 Canada; 6 Norwegian Ornithological Society, BirdLife Norway, Sandgata 30B,, 7012 Trondheim, Norway; 7 Maryland Department of Natural Resources, 580 Taylor Ave., Annapolis, MD 21401 USA; 8 High Arctic Institute, 603 10th Avenue, Orion, IL 61273 USA; 9 German Aerospace Center (DLR), German Remote Sensing Data Center (DFD), Münchenerstr. 20, 82234 Wessling, Germany; 10 Emirates Falconers’ Club, Al Mamoura Building (A), Muroor Road, P.O. Box, 47716, AbuDhabi, United Arab Emirates; 11 Faculty of Science, University of Alberta, 116 St NW, Edmonton, AB T6G 2R3 Canada; 12 Department of Biology and Centre d’études nordiques, Université Laval, 1045 avenue de la Médecine, Québec, QC G1V 0A6 Canada; 13 Department of Arctic Ecology, Norwegian Institute for Nature Research, Hjalmar Johansens gate 14, 9296 Tromso, Norway; 14 Kidd Biological Inc, 2911 Meridian Court, Anacortes, WA 98221 USA; 15 Division of Migratory Bird Management, U.S. Fish and Wildlife Service, 3000 Vintage Blvd 201, Juneau, AK 99801 USA; 16 Institute of Plant and Animal Ecology, Ural Division Russian Academy of Sciences, 8 marta str. 202/3, Yekaterinburg, Russia 620144; 17 Zoological Department, University of Agder, Universitetsveien 25 D, 4630 Kristiansand S, Norway; 18 Ned Smith Center for Nature and Art, 176 Water Company Rd, Millersburg, PA USA; 19 Department of Biology, University of Saskatchewan, 112 Science Place, Saskatoon, S7N 5E2 Canada; 20 Acopian Center for Conservation Learning, Hawk Mountain Sanctuary, 410 Summer Valley Road, Orwigsburg, PA 17961 USA

Correction to: *Scientific Reports* 10.1038/s41598-020-63312-0, published online 29 April 2020

The original version of this Article contained errors.

Andrew Dixon, Aleksandr Sokolov, Vasiliy Sokolov, David F. Brinker and Scott Weidensaul were omitted from the author list in the original version of this Article.

Ivan Pokrovsky was incorrectly affiliated with ‘Institute of Plant and Animal Ecology, Ural Division Russian Academy of Sciences, 8 marta str. 202/3, Yekaterinburg, 620144, Russia’. The correct affiliation is listed below:

Arctic Research Station of Institute of Plant and Animal Ecology, Ural Division Russian Academy of Sciences, 21 Zelenaya Gorka Str., Labytnangi, Yamal-Nenets autonomous district, 629400, Russia

In addition, the original version of this Article contained an error in Affiliation 9, which was incorrectly given as ‘German Aerospace Center (DLR), German Remote Sensing Data Center (DFD), Pfaffenwaldring 38–40, Stuttgart, 70569, Germany’. The correct affiliation is listed below:

German Aerospace Center (DLR), German Remote Sensing Data Center (DFD), Münchenerstr. 20, 82234 Wessling, Germany

The Acknowledgements section now reads:

We thank Risto Karvonen, Arve Østlyngen, Ken Gøran Uglebakken, Dag Gjerstad, Steve Aslaksen, Torkjell Morset, Jostein Sandvik and Ole Andreas Forseth for assistance with catching rough-legged buzzards in Norway. For assistance in the field with catching snowy owls in Norway, we thank Norwegian Nature Inspectorate (SNO) in Finnmark, Fjelltjenesten, Statskog (The State-owned Land and Forest Company) in Troms and Nordland. For helping to trap owls in Canada we thank Dan Zazelenchuk, Marten Stoffel, Mike Blom, Erhard Pletz, Mike Russel and for the help in the eastern part of Canadian Arctic, we thank Audrey Robillard. For assistance in trapping owls in the US we thank Mike Lanzone, Nova Mackentley, Tom McDonald, Chris Neri, Norman Smith, and Matt Solensky. We also thank all Project SNOWstorm (www.projectsnowstorm.org) supporters and collaborators. For fieldwork with rough-legged buzzards and peregrine falcons in Russia, we thank Olga Kulikova. For work on rough-legged buzzards in North America we thank Nina Kidd of Kidd Biological Inc, Travis Booms Alaska Dept of Fish and Game, Bryan Bedrosian of Teton Raptor Center, Neil Paprocki of Univ of Idaho - Moscow, Scott Thomas of Kidd Biological, Inc., Colin Dillingham USFS, Dylan Kesler of Vertebrate Systems LLC and Keith LaSage GeoTrak, Inc. We also thank Anne Scharf and Martina Scacco for the help with data analyses, participants of the IMPRS writing course for revising the manuscript and finally, to anonymous reviewers for constructive comments that substantially improved our manuscript.

Funding for the work on snowy owls in Norway was provided by the Norwegian Environment Agency, NOF-BirdLife Norway’s Snowy owl Foundation and the Environment departments at the Office of the County Governors of Troms and Finnmark, Trøndelag, Innlandet, Vestfold and Telemark, Oslo and Viken, Nordland and Vestland. Funding for the snowy owls in the eastern Canadian Arctic was provided by Polar Continental Shelf Project, the Garfield-Weston Foundation, Natural Science and Engineering Research Council of Canada, and Fonds Quebecois de Recherche en Nature et Technologies. The work on snowy owls in western Canada was funded by the Natural Sciences and Engineering Research Council of Canada (Grant 203177). Work with snowy owls in the US was funded by the Project SNOWstorm (www.projectsnowstorm.org). Funding for rough-legged buzzard work in North America was provided by Kidd Biological Inc, Alaska Department of Fish and Game and Arctic Raptors Inc and HWA Wildlife Consulting. Work on peregrine falcons in North America was funded by The Offield Family Foundation, Wolf Creek Charitable Foundation and The Peregrine Fund. Work on peregrine falcons in Yamal, Russia was funded by the Environment Agency-Abu Dhabi and coordinated by International Wildlife Consultants (UK) Ltd. The rest of the project was funded by the Max Planck Institute for Animal Behavior, Hawk Mountain Sanctuary, the Canada Research Chair program, and the Slovene Human Resources Development and Scholarship Fund. This study is part of the Ph.D. project of TC, with committee members KS, MW, NL, JFT, and IP.

The Author Contribution section now reads:

K.S., I.P., N.L., J.F.T., and M.W. participated in discussions and provided valuable feedback on the study design and data analyses. T.C. carried out the study. A.Die. provided snow cover data and performed interpolation of snow cover rasters to remove the effects of clouds and darkness. T.C. wrote the manuscript with significant input from K.S., I.P., N.L., J.F.T., and M.W. I.P., N.L., T.A., D.F.B., K.B., A.Dix., A.F., G.G., K.O.J., J.K., S.B.L., I.J.Ø., A.S., V.S., R.S., S.W., K.W., and J.F.T. shared tracking data and provided valuable comments on the manuscript. All authors read and approved the final manuscript for publication.

Lastly, in the Supplementary Information file originally published with this Article, additional data collection containing trapping methods was omitted.

These errors have been corrected in the PDF and HTML versions of the Article, and in the accompanying Supplementary Information file.

