# Supplementary material for: Arctic avian predators synchronise their spring migration with the northern progression of snowmelt
Source: Sci Rep. 2020 Apr 29;10:7220. doi: 10.1038/s41598-020-63312-0 (PMC7190624; doi:10.1038/s41598-020-63312-0)
Supplement: Supplementary file 1 — Supplementary material. [file 41598_2020_63312_MOESM1_ESM.pdf]

Supplementary material: Arctic avian predators synchronise their spring migration with the northern progression of snowmelt

Teja Curk[1,2*], Ivan Pokrovsky[1,3,4], Nicolas Lecomte[5], Tomas Aarvak[6,+], Kurt Burnham[7,+], Andreas Dietz[8,+], Alastair Franke[9,+], Gilles Gauthier[10,+], Karl-Otto Jacobsen[11,+], Jeff Kidd[12,+], Stephen B. Lewis[13,+], Ingar J. Øien[6,+], Roar Solheim[14,+], Karen Wiebe[15,+], Martin Wikelski[1,2], Jean-François Therrien[16], Kamran Safi[1,2]

[1]Max Planck Institute of Animal Behavior, Department of Migration, Am Obstberg 1, Radolfzell, 78315, Germany

[2]University of Konstanz, Department of Biology, Universitätsstraße 10, Konstanz, 78464, Germany

[3]Institute of Plant and Animal Ecology, Ural Division Russian Academy of Sciences, 8 marta str. 202/3, Yekaterinburg, 620144, Russia

[4]Institute of Biological Problems of the North, Magadan, Ulitsa Portovaya 18, 685000, Russia

[5]Canada Research Chair in Polar and Boreal Ecology, Department of Biology, Université de Moncton, 18 Antonine-Maillet, Moncton, NB E1A 3E9, Canada

[6]Norwegian Ornithological Society, BirdLife Norway, Sandgata 30B, Trondheim, 7012, Norway

[7]High Arctic Institute, 603 10th Avenue, Orion, IL 61273, USA [8]German Aerospace Center (DLR), German Remote Sensing Data Center (DFD), Pfaffenwaldring 38-40, Stuttgart, 70569, Germany

[9]University of Alberta, Faculty of Science, 116 St NW, Edmonton, AB T6G 2R3, Canada

[10]Université Laval, Department of Biology and Centre d'études nordiques, 1045 avenue de la Médecine, Québec, QC G1V 0A6, Canada

[11]Norwegian Institute for Nature Research, Department of Arctic Ecology, Hjalmar Johansens gate 14, Tromso, 9296, Norway

[12]Kidd Biological Inc, Anacortes, WA 98221, USA [13]U.S. Fish and Wildlife Service, Division of

Migratory Bird Management, 3000 Vintage Blvd 201, Juneau, AK 99801, USA
[14]University of Agder, Zoological Department, Universitetsveien 25 D, Kristiansand S, 4630, Norway
[15]University of Saskatchewan, Department of Biology, 112 Science Place, Saskatoon, S7N 5E2, Canada
[16]Hawk Mountain Sanctuary, Acopian Center for Conservation Learning, 410 Summer Valley Road, Orwigsburg, PA 17961, USA
[*]Correspondence: tcurk@ab.mpg.de
[+]these authors contributed equally to this work

Data collection

Snowy owls in western Canadian Arctic

Locations: Areas between Kyle and Saskatoon (Saskatchewan, Canada), Peace River area (Alberta, Canada)

Capture methods: Bal-chatri, remote control released bow-net trap or bal-chatri trap with pigeon or hamster as a lure during wintering

Transmitters used: GPS–GSM transmitters (Ecotone Telemetry, model Saker H, 25 g), Geo-Trak 30g battery ARGOS PTT, Microwave 30g GPS Solar ARGOS PTT

Permits: Animal Care permit from the University of Saskatchewan (number 20090025); banding permits 10736 and 10737

Snowy owls in eastern Canadian Arctic

Locations: Bylot Island, Nunavut in 2007 (n = 12) and 2014 (n = 10), Mary River, Nunavut in 2011 (n = 1) and Deception Bay, Nunavik, QC in 2013

Capture methods: Bow-nets positioned over the nest and bal-chatri

Transmitters used: ARGOS satellite transmitters (Microwave telemetry, USA, PTT-100, n = 14; North Star Science and Technology, LLC, USA, PTT-30G, n = 17)

Permits: Work was conducted under approval from CPAUL (Comite de Protection des Animaux de l'Universite Laval)

Snowy owls in Norway

Locations: Troms and Finnmark County

Capture methods: Bow-net (2011 and 2015), and metal grid with multiple nooses (2007)

Transmitters used: Microwave Telemetry 35 g solar PTT (2 females), Microwave 35 g battery powered PTT (2007), Microwave Telemetry 30 g solar GPS PTT (3 ind.), Geo-Trak 30 g battery PTT (2011), Microwave Telemetry 30 g solar GPS PTT (4 ind.), Geo-Trak 30 g battery PTT (2015)

Permits: Permission for catching and instrumenting snowy owls in Norway provided from Animal welfare unit in Norway: 2007-2008 (FOTS ID 209), 2011-2012 (FOTS ID 3346), 2015-2016 (FOTS ID 7561) and from Norwegian Environment Agency: 2007 (2007-6268 ART-VI-ARES), 2011-2015 (2011-3906 ART-VI-ORD).

Rough-legged buzzards in North America

Locations: Rankin Inlet, Nunavut (Canada); Peace River, Alberta (Canada); Alaska, California Nevada, Washington, Idaho, Utah, Montana, Wyoming (USA)

Capture methods: Bal-chatri with house mouse *(Mus musculus)*

Transmitters used: Ecotone 22g solar GPS GSM saker H, Microwave telemetry inc 22 and 30g GPS ARGOS PTT, Geotrak 22g GPS argos PTT

Permits: USGS Bird Banding Lab Master Permit number 22951

Rough-legged buzzards and peregrine falcons in Russia

Locations: Kolguev Island (rough-legged buzzard and peregrine falcon), Nenetsky Ridge (rough-legged buzzard), Vaigach Island (rough-legged buzzard)

Capture methods: Bow-net and Clap-Net Trap on the nest

Transmitters used: E-obs GPS-GSM 43 g for the rough-legged buzzard and E-obs GPS-GSM 25 g and University of Konstanz GPS-GSM 15 g for both rough-legged buzzard and peregrine falcon.

Permits: No specific permissions were required according to the §44 and §6 of the Federal Law of the Russian Federation No. 52 from 24.04.1995 (last update 03.08.2018) “On Wildlife” to carry out the work for this study. There were no Special Protected Natural Territories in our study area, and our activities did not include withdrawal of investigated species from nature. In Nenetsky, the work was carried out in agreement with the Nenetsky Nature Reserve in a buffer zone.

Peregrine falcons in North America

Locations: Centered around the Thule Air Base area (within 100 km), northwest Greenland (76.5° N, 68.7° W)

Capture methods: Harnessed pigeon, noose carpets, and Dho-gaza net

Transmitters used: Microwave Telemetry, 20g Argos PTT, North Star Telemetry, 20g Argos PTT

Permits: Permit to conduct scientific research in Greenland issued by the Government of Greenland and The Danish Polar Center

Determination of spring migration periods

We determined spring migration periods for each individual track using First Passage Time (FPT) following [1] and [2]. FPT is the time it takes for an animal to cross a circle of a given radius [3]. Here, low FPT values correspond to travelling between the wintering and breeding sites, while high FPT values correspond to staying on the wintering or the breeding site. For calculating FPT, we used R package "adehabitatLT" [4]. First, we empirically assessed the radii sizes separately for each species following [1]. We assessed 100 radius values ranging from 1000 to 10000 meters. For each radius and migration track, we calculated the FPT variance and then we calculated the mean variance per radius. We used the maximum value of the log-transformed mean FPT variance per track plotted against radius to select the radius value (one value per species) in the calculations of FPT of each individual track. Second, we segmented FPT profiles (plotted FPT values against the time of each migration track) by identifying changes in mean and variance using R package "changepoint" [5]. We then segmented tracks to migration and breeding periods at the breakpoints of mean and variance of FPT profiles. We classified periods of FPT less than five days as travelling, between five and 30 days as searching and more than 30 days as stationary. We defined spring migration periods between the first and last day as travelling, before searching or settling at the breeding grounds. Individual FPT profiles were inspected manually to assure the relevant selection of spring migration periods.

References

[1] Fauchald, P. & Tveraa, T. Using first-passage time in the analysis of area-restricted search and habitat selection. *Ecology* **84**, 282–288 (2003).

[2] Le Corre, M., Dussault, C. & Côté, S. D. Detecting changes in the annual movements of terrestrial migratory species: using the first-passage time to document the spring migration of caribou. *Mov. Ecol.* **2**, 19 (2014).

[3] Johnson, A., Milne, B. & Wiens, J. Diffusion in fractcal landscapes: simulations and experimental studies of tenebrionid beetle movements. *Ecology* **73**, 1968–1983 (1992).

[4] Calenge, C. The package "adehabitat" for the r software: a tool for the analysis of space and habitat use by animals. *Ecol. Modell.* **197**, 516–519 (2006).

[5] Killick, R. & Eckley, I. Changepoint: an r package for changepoint analysis. *J. Stat. Softw.* **58**, 1–19 (2014).

[6] Fieberg, J. R. *et al.* Used-habitat calibration plots: a new procedure for validating species distribution, resource selection, and step-selection models. *Ecography* **41**, 737–752 (2018).

Table S1: Binomial mixed models with snow cover as a dependent variable, interaction between day and species as a predictor variable, and individual or year as a random effect. To evaluate the effect of the predictor, we compared a model with and without species included as predictor. Only the results of the full models are presented and those with lower AIC and ΔAIC >2 are marked with *.

**Dep.var. n = number of locations)**	**Fixed eff.**	**Random eff.**	**Est.**	**SE**	**z value**	**p value**	**ΔAIC**	**AIC Weight**	**LL**
Snow cover n = 207608	(Intercept)	Individual	-3.45	0.53	-6.55	<0.001	42*	1.0	-108111
	Day		-0.13	0.01	-10.85	<0.001			
	Spec. RL		2.37	0.56	4.20	<0.001			
	Spec. SO		4.86	0.59	8.23	<0.001			
	Day:Spec. RL		0.03	0.01	2.80	0.004			
	Day:Spec. SO		0.03	0.01	2.91	0.005			
Snow cover n = 207608	(Intercept)	Year	-2.33	0.46	-5.06	<0.001	23526*	1.0	-116280
	Day		-0.13	0.01	-11.64	<0.001			
	Spec. RL		2.98	0.97	39.60	<0.001			
	Spec. SO		4.43	0.08	58.68	<0.001			
	Day:Spec. RL		0.05	0.01	4.12	<0.001			
	Day:Spec. SO		0.05	0.01	4.45	<0.001			

Table S2: Movement decisions of the snowy owl and rough-legged buzzard according to snow cover at three- and five-day step length. We performed mixed conditional logistic regression models with movement choice (used vs alternative locations) as a dependent variable, snow cover as a predictor and stratum nested in individual as a random effect. We performed models separately by species. To evaluate the effect of each predictor, we compared models with and without the predictor. Only the results of the full models are presented.

**Step lengt**	**Species (n = number of locations)**	**Pred.**	**Coef.**	**SE**	**z value**	**p value**	**ΔAIC**	**AIC Weight**	**LL**
Three-day	Snowy owl n = 15323	Snow cover	-3.45	0.53	-6.55	0.30	1	0.4	-11986
Three-day	Rough-legged buzzard n = 22583	Snow cover	0.01	0.05	0.27	0.80	2	0.3	-20300
Three-day	Peregrine falcon n = 1276	Snow cover	0.28	0.38	0.75	0.47	1	0.3	-693
Five-day	Snowy owl n = 8316	Snow cover	-0.03	0.09	-0.36	0.72	2	0.3	-5836
Five-day	Rough-legged buzzard n = 12100	Snow cover	0.05	0.07	0.76	0.45	1	0.3	-10166
Five-day	peregrine falcon n = 550	Snow cover	-0.08	1.33	-0.06	0.95	2	0.3	-277

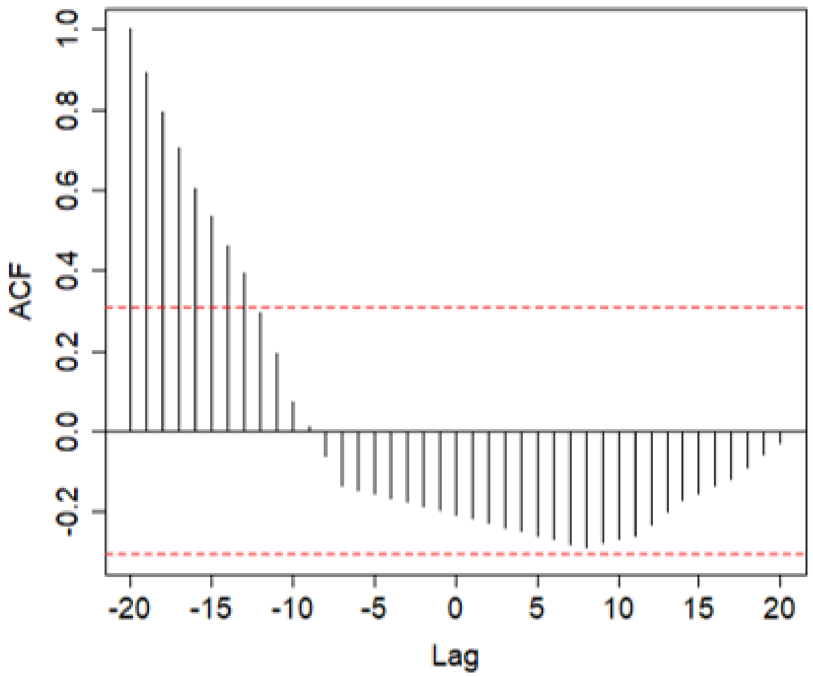

Figure S1: Auto-Correlation Function (ACF) of snow cover at time-lag -20 to 20 days for a rough-legged buzzard on May 12, 2016. Red lines represent 95% confidence interval. Note that auto-correlation is only significant for -20 to approximately -10 days.

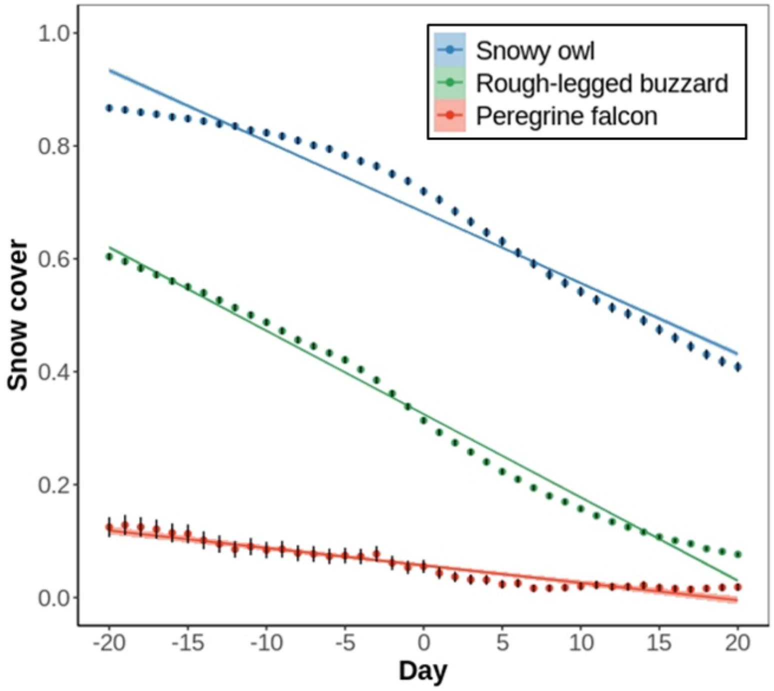

Figure S2: Snow cover conditions (0 = snow absence; 1 = snow presence) at each of the birds' position compared between the days (from 20 days in the past to 20 days in the future). Dots with lines represent mean ±SE of the raw data and shaded areas represent SE of the model estimates.

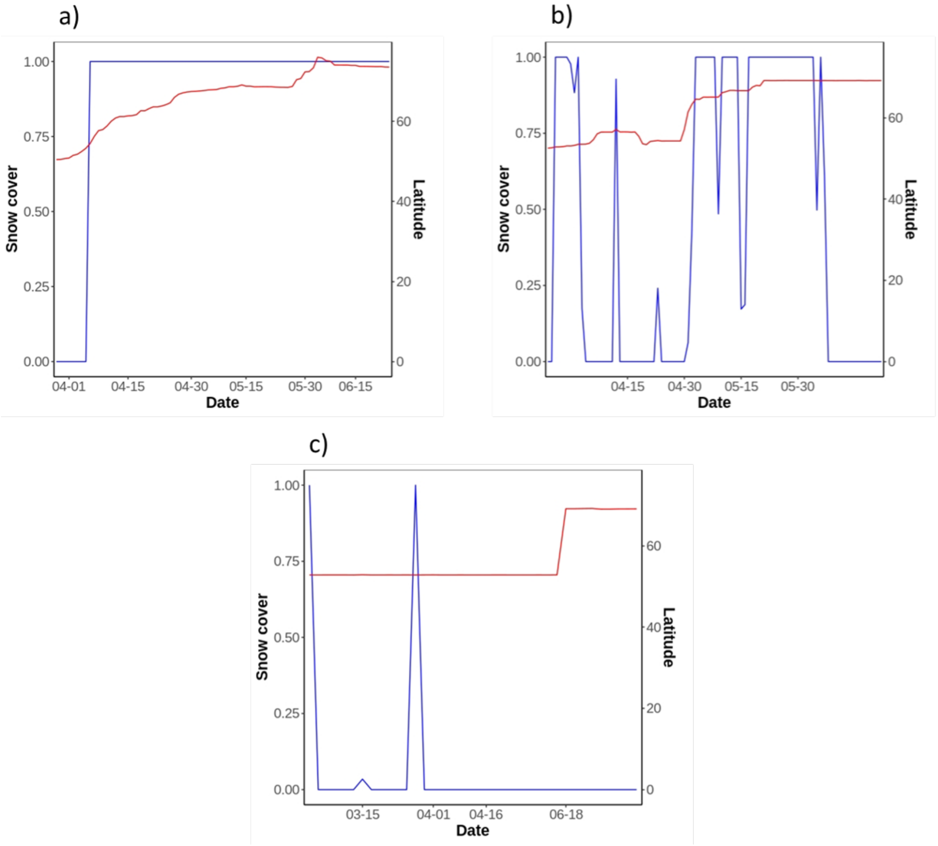

Figure S3: The individual response of the snowy owl (a), rough-legged buzzard (b) and the peregrine falcon (c) to the snow cover when moving northward during spring migration.

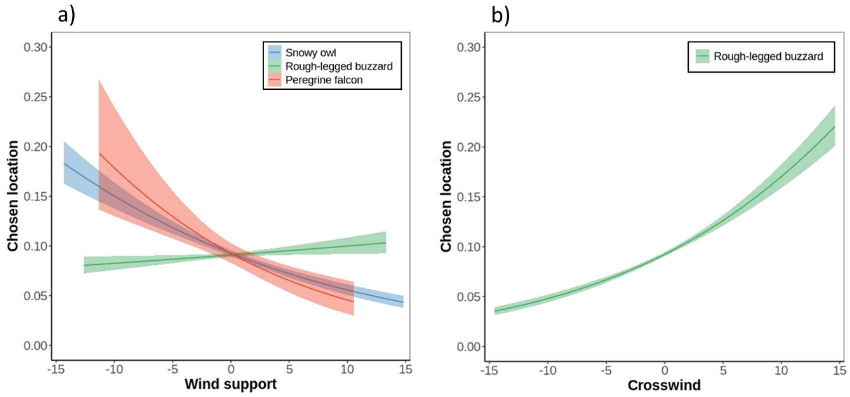

Figure S4: Mixed conditional logistic regression models estimating (a) the impact of wind support and (b) the impact of crosswind (m/s) on movement decisions (0-alternative vs 1-used locations) at one-day step length (Table 3). Shaded areas represent standard error. Shaded areas represent SE of the model estimates.

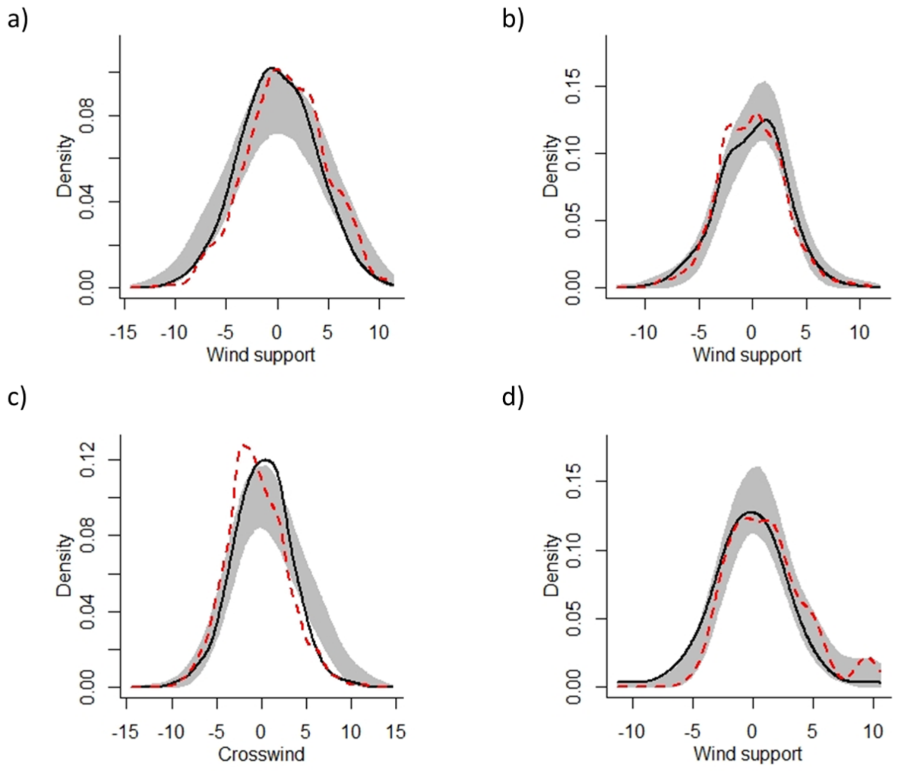

Figure S5: Validation of SSF using UHC plots following [6]. Only plots for predictors that significantly influenced movement decisions (Table 3, Table S2) are presented. (a) the snowy owl, (b) and (c) the rough-legged buzzard and (d) the peregrine falcon. The black line represents the distribution of the environmental variable at used locations, the grey area represents the UHC simulation envelope, and the red line represents the distribution of alternative locations in SSF.
